# Embracing Responsible Leadership and Enhancing Organizational Citizenship Behavior for the Environment: A Social Identity Perspective

**DOI:** 10.3389/fpsyg.2021.632629

**Published:** 2021-09-29

**Authors:** Xiaohong Xiao, Zheng Zhou, Fu Yang, Huijie Qi

**Affiliations:** School of Business Administration, Guizhou University of Finance and Economics, Guiyang, China

**Keywords:** responsible leadership, moral identity, OCBE, individualism, social identity

## Abstract

Although organizational citizenship behavior for the environment (OCBE) literature has highlighted the critical role of leadership on the emergence of OCBE, there is still room for further research exploration of how and when leaders influence subordinates’ OCBE. According to social identity theory, we propose a theoretical model that responsible leadership promotes subordinates’ OCBE by examining subordinates’ moral identity as a mediator and individualism as a boundary condition. Using a sample of 273 collected in China, results indicated that responsible leadership was positively related to subordinates’ moral identity, which in turn was positively related to subordinates’ OCBE. Subordinates’ moral identity partially mediated the relationship between responsible leadership and their OCBE. In addition, both the relationship between responsible leadership and subordinates’ moral identity and the indirect relationship between responsible leadership and subordinates’ OCBE were stronger when individualism was lower. These findings provide novel insights into how responsible leadership influences OCBE and how such influence is shaped by subordinates’ individualism.

## Introduction

Organizational citizenship behavior for the environment (OCBE) is defined as discretionary activities by subordinates within the organization that are not rewarded or required and are directed toward environmental improvement (Daily et al., 2008). Effective implementation of OCBE can bring significant value to the success of companies, such as reducing the waste of personal and organizational resources (Hanna et al., 2000), enhancing the success of organizational environmental performance in an ever-changing marketplace, and promoting companies’ sustainable development (Boiral and Paillé, 2012). Indeed, given that subordinates are the agents who implement the organizational environmental policies (Dumont et al., 2017), and the success of environmental programs often depends on subordinates’ behaviors (Robertson and Barling, 2013), researchers have begun to investigate the factors that can affect OCBE, such as megaproject environmental responsibility (Wang et al., 2017), institutional pressures (Wang et al., 2018), environmental management practices (Paillé et al., 2013a), environmental intent (Poortinga et al., 2004), strategic human resource management (Paillé et al., 2013b), and organizational support (Paillé and Boiral, 2013).

With promising progress in the OCBE literature, a large number of scholars have begun to focus on how leadership style influences subordinates’ OCBE (e.g., Afsar et al., 2016; Han et al., 2019; Mi et al., 2019), because supervisors’ support for environmental efforts plays a key role in the emergence of OCBE (Choi, 2007; Ramus and Killmer, 2007; Daily et al., 2008; Paillé et al., 2013a). In addition, previous research has shown that leaders serve as role models and their environmental values can influence the environmental motivations and behaviors of their followers (Derue et al., 2011; Kim et al., 2017). Consistent with this line of research (e.g., Han et al., 2019), this study focuses on responsible leadership as an important antecedent of OCBE, given that the characteristics of responsible leadership are paying attention to the impact of the organizational environment on stakeholders, involving subordinates in the environmental decision-making process, and providing support (Maak and Pless, 2006; Maak, 2007; Voegtlin et al., 2012; Temminck et al., 2013) that are consistent with the values of OCBE. Further, previous research has indicated that subordinates are less likely to proactively implement unrewarded and unrequested environmental behaviors in an organization unless they have higher moral identity (Rupp et al., 2013; May et al., 2015). Hence, a potential inadequacy of the current research is neglect of the fact that moral identity may be considered an important potential explanatory mechanism to denote the relationship between responsible leadership and OCBE. Indeed, responsible leadership emphasizes ethical norms and values in an organization (Maak and Pless, 2006; Maak, 2007), which is helpful to enhancing subordinates’ moral cognition and inspiring their moral identity, thereby effectively promoting the generation of OCBE (Rupp et al., 2013). Correspondingly, according to social identity theory (Tajfel and Turner, 1979; Aquino and Reed, 2002), when individuals recognize that they belong to a specific social group, they actively develop a social identity with the organization and maintain their inter group identity, which in turn helps to align their attitudes and behaviors congruent with the behavioral norms, values and goals advocated by the organization. Therefore, social identity theory is used to examine responsible leadership as a key predictor of OCBE and to identify moral identity as an important mediating mechanism linking responsible leadership to OCBE.

However, responsible leadership does not have the same effects in all situations. The globalization of the world economy, the development of multinational corporations, and the penetration of cultural values in western countries have caused Chinese cultural values to gradually change, including the pursuit of freedom and competition and an increase in divorce rates, which are reflected in the emergence of individualism (e.g., Kashima et al., 2011). Examining the impact of individualism is important to understand the changes in Chinese cultural values and effectively respond to the challenges posed by such changes. Indeed, previous research has demonstrated significant differences in individuals’ attitudes and behaviors in different cultural values situations (Wagner, 1995; Ilies et al., 2007). Subordinates with strong individualism emphasize that they are independent of others, act on personal values, and focus on personal goals (Ilies et al., 2007; Ng et al., 2011). By contrast, those with weak individualism attach importance to stay consistent with the values of leaders and organizations and expect to achieve the team’s common goals by participating in decision making (Wagner, 1995; Dickson and Weaver, 1997). This study adopts the cultural value perspective and further argues that individualism may limit the impact of responsible leadership because subordinates with strong individualism are guided by their personal attitudes rather than external factors, such as their supervisors (Hooft and Jong, 2009). Consequently, this research uses individualism as a crucial boundary condition to understand the circumstances under which responsible leadership is strengthened or weakened.

**FIGURE 1:**
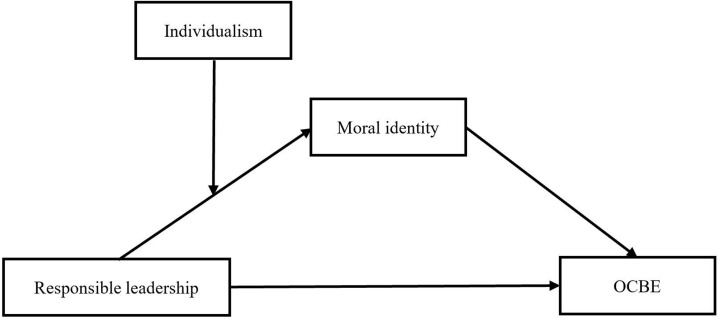
Theoretical model. OCBE, organizational citizenship behavior for the environment.

Taken together, this research makes several important contributions to the literature. First, based on social identity theory (Tajfel and Turner, 1979; Aquino and Reed, 2002), moral identity is cast to explain the relationship between responsible leadership and OCBE, which provides a novel perspective on how responsible leadership enhances OCBE and responds to the call to explore the bridge that links responsible leadership with subordinate behavior (Doh and Quigley, 2014). Second, this study provides new insights into when responsible leadership is effective or ineffective for OCBE on the basis of the cultural value perspective by introducing individualism as an important boundary condition for the effects of responsible leadership. Third, our research provides a clear picture to understand how responsible leadership influences OCBE and how such influence is shaped by the individualism of subordinates. Figure 1 depicts the overall theoretical model.

## Present Investigation

In the current research, based on social identity theory, we argue that moral identity as an important mediating mechanism linking responsible leadership to OCBE. Furthermore, this research uses individualism as a crucial boundary condition to understand the circumstances under which responsible leadership is strengthened or weakened. Accordingly, we focused on the mediating role of moral identity as previous research has indicated that subordinates are less likely to proactively implement unrewarded and unrequested environmental behaviors in an organization unless they have higher moral identity (Rupp et al., 2013; May et al., 2015). In addition, based on cultural value perspective, we are concerned about the moderating role of individualism because previous research has demonstrated significant differences in individuals’ attitudes and behaviors in different cultural values situations (Wagner, 1995; Ilies et al., 2007).

Prior to presenting the hypothesized model, it is important to note how the present investigation differs from, and extends, the work of previous related research (e.g., Robertson and Barling, 2013; Han et al., 2019). Han et al. (2019) research investigated the mediating effect of felt obligation for constructive change on the relationship between responsible leadership and OCBE, whereas the current research examines moral identity as an important mediating mechanism linking responsible leadership to OCBE. Indeed, previous research has yet to determine whether leaders influence subordinates’ OCBE by shaping their moral identity, hence we extended the research of the underlying psychological processes and mechanism though which leaders motivate subordinates’ OCBE. Additionally, as noted by Han et al. (2019), social cognitive theory has been used as a theoretical basis to explain the influence of responsible leadership on subordinates’ OCBE. In comparison, based on social identity theory (Tajfel and Turner, 1979; Aquino and Reed, 2002), when individuals recognize that they belong to a specific social group, they actively develop a social identity with the organization and maintain their inter group identity, which in turn helps to align their attitudes and behaviors congruent with the behavioral norms, values and goals advocated by the organization. Therefore, the current research provides a novel perspective on how responsible leadership enhances subordinates’ OCBE. Finally, Han et al. (2019) examined the moderating role of supervisor-subordinate guanxi, whereas the current research extends the current understanding of the boundary conditions under which responsible leadership is effective or ineffective, by demonstrating individualism as a key moderator.

Similarly, Robertson and Barling (2013) developed and tested a model that links environmentally-specific transformational leadership and leaders’ workplace pro-environmental behaviors to employees’ pro-environmental passion and behaviors, whereas we propose a theoretical model that responsible leadership promotes subordinates’ OCBE by examining subordinates’ moral identity as a mediator and individualism as a boundary condition, thereby providing a clear picture for understanding how responsible leadership influences OCBE and how such influence is shaped by subordinates’ individualism. Specifically, first, Robertson and Barling (2013) research investigated the effects of environmentally specific transformational leadership and leaders’ environmental behaviors on employees’ environmental behaviors, whereas the current research focuses on the effects of responsible leadership on subordinates’ OCBE. Second, as noted by Robertson and Barling (2013), social learning theory was used to illustrate the relationship between leaders’ environmental behaviors and employees’ environmental behaviors. However, as discussed above, based on social identity theory, the current research examines moral identity as an important mediating mechanism, which provides a novel perspective on how leadership style influences subordinates’ OCBE. Third, compared with Robertson and Barling (2013) research, the current research uses individualism as a crucial boundary condition, which provides a new insight into comprehending how to improve the effectiveness of responsible leadership and answers the important question of a call for investigating the influence of individualism cultural values in an organization (Hooft and Jong, 2009; Santos et al., 2017).

## Theory and Hypotheses

### Responsible Leadership and Subordinates’ Organizational Citizenship Behavior for the Environment

In a global stakeholder society, leaders are not only required to be responsible for a company’s financial performance but also for the economic, environmental, and societal impacts of stakeholders (Maak and Pless, 2006; Voegtlin, 2011). Responsible leadership can be defined as “the art and ability involved in building, cultivating and sustaining trustful relationships to different stakeholders, both inside and outside the organization, and in coordinating responsible action to achieve a meaningful, commonly shared business vision” (Maak, 2007, p. 334). Responsible leadership emphasizes the leader’s responsibility to a multitude of stakeholders (Pless, 2007), advocates subordinates’ ethical behavior and participation in decision making (Maak and Pless, 2006), which ensures that production processes meet environmental requirements (Han et al., 2019), and establishes public trust and promotes the company’s sustainable development (Doh and Quigley, 2014).

Subordinates’ attitudes and behavior in the organization are often influenced by leadership characteristics. Responsible leadership is asserted to facilitate the promotion of subordinates’ OCBE. First, responsible leadership attaches importance to the impact of corporate decision making on society and the natural environment and takes responsibility for environmental stakeholders in the form of corporate social responsibility (Maak and Pless, 2006). Indeed, responsible leadership establishes strict environmental protection standards to assist subordinates in believing that corporate social responsibility is an important theme in the organization (Waldman and Siegel, 2008). Therefore, when the corporate environment is threatened, subordinates actively seek solutions and maintain the corporation’s sustainable development (Dirks and Ferrin, 2002). In addition, responsible leadership adopts the fulfillment of corporate social responsibility as the goal of organizational development (Pless, 2007), which helps subordinates realize that corporate social responsibility activities are part of their daily activities and further strengthens their willingness to fulfill their corporate social responsibility (Voegtlin et al., 2012) through activities such as saving organizational resources and protecting the organizational environment.

Second, responsible leadership establishes an ethical corporate culture in the organization to assist corporations in building public trust and maintaining good social images (Voegtlin et al., 2012), such leaders are arguably guardians of moral and environmental values (Maak and Pless, 2006). In general, responsible leadership appreciates subordinates who contribute to the maintenance of the corporate environment (Cameron, 2011), thereby providing organizational support to motivate subordinates to engage in ethical behavior (Maak and Pless, 2006). In return for the organization’s support, subordinates engage in organizational citizenship behavior that is conducive to the corporate environment and consistent with the corporation’s environmental values.

Third, responsible leadership actively interacts with subordinates and involves them in decision-making processes (Doh and Quigley, 2014), which helps reduce subordinates’ unethical behavior. Specifically, this method of solving difficult ethical problems in the organization through consensus enables finding solutions that are satisfactory to all team members, thus effectively reducing the possibility of unethical behavior (Voegtlin, 2011). Moreover, participatory decision making generates stronger work motivation and organizational citizenship awareness (Podsakoff, 2000; Haque et al., 2018) when subordinates believe that they can provide positive contributions to leadership and organizational decision making (Voegtlin et al., 2012). Thus, the implementation of ethical behavior in the organization is further strengthened. As a result, responsible leadership is hypothesized as being positively related to subordinates’ OCBE.

Hypothesis 1: Responsible leadership is positively related to subordinates’ OCBE.

### The Mediating Role of Moral Identity

Moral identity is defined as “an individual difference reflecting the degree to which being moral is central or characteristic of a person’s sense of self” (Mulder and Aquino, 2013, p. 220), which is generally influenced by the moral values of leaders and the organization (May et al., 2015). Responsible leadership is proposed to have a direct and significant impact on subordinates’ moral identity. First, by definition, to present a good corporate image and sustainable development, responsible leadership regards that environmental stakeholders must adopt corporate social responsibility (Maak and Pless, 2006). In fact, if subordinates believe that the organization engages in corporate social responsibility and behaves in a manner that is consistent with its values, they are more likely to have a sense of identity and choose to join the organization. Additionally, responsible leadership is a positive role model for citizenship behavior because they consider the consequences of corporate decision making on environmental stakeholders and incorporate the benefits of environmental stakeholders in decision situations (Voegtlin et al., 2012), which motivates subordinates to learn from such appealing leadership behavior and further enhances their moral identity (Doh and Quigley, 2014).

Second, responsible leadership creates a positive moral and cultural atmosphere for subordinates in the organization and improves their moral identity. Specifically, responsible leadership transforms abstract ethical concepts into specific corporate moral standards to assist subordinates in establishing moral values and further enhancing their moral identity in the organization (Cameron, 2011; Antunes and Franco, 2016). In addition, when subordinates abide by moral concepts and rules in their work, they can gain more recognition and organizational support from leaders (Maak, 2007; Pless, 2007). Thus, to repay leaders and organizations, subordinates pay more attention to organizational moral standards and further improve their moral identity (Grant, 2012).

Third, by involving subordinates in the organizational decision-making process, responsible leadership can enhance subordinates’ moral identity. Indeed, participatory decision making creates an open and free working environment, enhancing subordinates’ awareness to monitor and solve the organization’s moral problems (Voegtlin et al., 2012), which is conducive to forming their moral identity. Moreover, responsible leadership focuses on the views of different stakeholders, thus involving subordinates in organizational decision making (Maak and Pless, 2006). Logically, then, when subordinates perceive that leaders attach great importance to their own views, they will believe that leaders are more visionary and resonate psychologically at the individual level (Doh and Quigley, 2014), thus enhancing their moral identity. Hence, responsible leadership is hypothesized to be positively related to subordinates’ moral identity.

Hypothesis 2: Responsible leadership is positively related to subordinates’ moral identity.

In addition, the motivation of subordinates to implement OCBE is argued to have relevance for moral identity. First, compared with subordinates with weak moral identity, subordinates with a strong moral identity regard moral values as the center of self-definition (Aquino and Reed, 2002), which helps promote the implementation of OCBE. Specifically, subordinates with a strong moral identity attach importance to their ethical behavior in the organization (May et al., 2015), because they are more concerned about upholding a moral self-image (Rupp et al., 2013). As such, subordinates with a strong moral identity tend to compare their behavior with existing organizational moral standards and adjust their behavior when they fall short of these standards (Reed and Aquino, 2003; Mulder and Aquino, 2013), thereby promoting the implementation of OCBE.

Second, subordinates with a strong moral identity pay more importance to the organization’s moral values than those with weak moral identity (Ashforth and Mael, 1989) and maintain the corporate social image by implementing positive organizational citizenship behaviors, such as prosocial behaviors (Mulder and Aquino, 2013). Furthermore, subordinates with a strong moral identity have a strong sense of organizational belonging, which further strengthens their positive attitude toward the organization’s ethical activities (May et al., 2015), leading to reduce unethical behavior in the organization. Hence, subordinates with a strong moral identity are more motivated to engage in behaviors that are consistent with the organization’s moral values (Aquino et al., 2007, 2009), thereby increasing the implementation of OCBE. Consequently, subordinates’ moral identity is hypothesized to be positively related to subordinates’ OCBE.

Hypothesis 3: Subordinates’ moral identity is positively related to subordinates’ OCBE.

Thus far, this study proposes that subordinates’ moral identity captures an important mechanism through which responsible leadership is positively related to their OCBE. According to social identity theory (Tajfel and Turner, 1979), individual moral identity is strongly influenced by moral situations, and the individual’s identification with the group and its common norms will form a positive moral climate, which in turn will promote the improvement of individual moral identity (Aguinis and Glavas, 2017). Indeed, subordinates holding strong moral identity are likely to strengthen the consistency between their moral selves and behaviors (Ashforth and Mael, 1989; May et al., 2015), as moral identity is a potential social identity that may be a part of a person’s social self-schema (Tajfel, 1982; Aquino and Reed, 2002). Hence, when responsible leadership helps subordinates shape and improve their moral identity, they regard moral values as the center of self-definition and are more motivated to engage in behaviors that are consistent with organizational moral values (Aquino and Reed, 2002; Aquino et al., 2007, 2009), thus further enhancing the implementation of OCBE. Taken together, based on Hypothesis 2 and 3, we propose a mediating role of subordinates’ moral identity in transmitting the effect of responsible leadership on their OCBE.

Hypothesis 4: Subordinates’ moral identity mediates the relationship between responsible leadership and subordinates’ OCBE.

### The Moderating Role of Individualism

As an important part of cultural values, individualism describes how an individual views the relationship between himself or herself and the collective (Hofstede, 1984). Subordinates with high individualism emphasize that they are independent of others, act on personal values, and focus on personal goals (Ilies et al., 2007; Ng et al., 2011). By contrast, those with low individualism attach importance to maintain consistency with the values of leaders and the organization and expect to achieve the common goals of the team by participating in decision making (Wagner, 1995; Dickson and Weaver, 1997). Hence, we expect that responsible leadership will not be equally effective under different degrees of individualism in promoting subordinates’ moral identity.

First, subordinates with high individualism pursue the maximization of their interests as their personal goals (Wagner, 1995) rather than upholding environmental stakeholders’ interests. Indeed, responsible leadership views upholding environmental stakeholders’ interests as the corporation’s goal (Maak and Pless, 2006), which may be inconsistent with the goals of subordinates with high individualism. In such cases, this discrepancy will become a burden that subordinates with high individualism must bear in achieving their individual goals (Earley, 1989), thereby weakening the impact of responsible leadership on subordinates’ moral identity. In contrast, subordinates with low individualism are more motivated to achieve the goal of upholding the interests of environmental stakeholders, as they pay more attention to the consistency between personal and corporate goals (Dickson and Weaver, 1997). Thus, this consistency reinforces the relationship between responsible leadership and subordinates’ moral identity.

Second, compared with subordinates with low individualism, subordinates with high individualism follow their values rather than leadership and organizational values (Morris et al., 1993). When subordinates with high individualism interact with responsible leadership, they may conflict because of the misfit between individual values and the values that the leaders or organizations established (Hooft and Jong, 2009), which decreases subordinates’ moral identity with responsible leadership. Conversely, as subordinates’ degree of individualism decreases, their cognition is more guided by the organization’s explicit or implicit group social norms (Ilies et al., 2007), thus resulting in a better fit with the values of leaders or organizations. At the same time, having values that fit with those of responsible leadership enhances subordinates’ resonance, and they will show stronger moral identity to responsible leadership.

Third, subordinates with high individualism emphasize that they are independent of others (Ng et al., 2011), weakening their moral identity to responsible leadership. Specifically, subordinates with high individualism pay more attention to self-sufficiency and control (Morris et al., 1993) and obtain satisfaction from and are proud of their self-achievement (Earley, 1989). When participatory decision making violates the willingness of subordinates with high individualism to maintain independence, they choose to confront team members or even withdraw from the organization, thereby weakening their moral identity. In contrast, subordinates who lack individualism demonstrate to responsible leadership that they are consistent with the organization’s activities by participating in organizational decision making (Earley, 1993). Hence, such consistency between subordinate attitude and leadership intention reinforces the long-term cooperative relationship between subordinates and leaders and further enhances subordinates’ moral identity to responsible leadership. Taken together, we propose a moderating role of individualism in the relationship between responsible leadership and subordinates’ moral identity.

Hypothesis 5: Individualism moderates the relationship between responsible leadership and subordinates’ moral identity, such that the relationship is weaker when individualism is high rather than low.

### Integrated Model

To integrate these relationships, we propose a moderated mediation model in which individualism moderates the indirect relationship between responsible leadership and subordinates’ OCBE. From the perspective of cultural values, subordinates with high individualism pay attention to the realization of personal goals, follow self-values, and are independent of other members of the organization, which may be inconsistent with the corporate goals and organizational values advocated by responsible leadership. Such inconsistency should make responsible leadership ineffective in promoting subordinates’ moral identity. As a result, subordinates’ moral identity plays a less important role in transmitting the effect of responsible leadership on subordinates’ OCBE.

In contrast, subordinates with low individualism are more motivated to choose to be consistent with the goals and values of their leaders or organization, thus responsible leadership is more effective in helping subordinates to shape and improve moral identity. Subordinates’ moral identity then plays a more important role in mediating the effect of responsible leadership on subordinates’ OCBE. Taken together, we propose that individualism moderates the indirect relationship between responsible leadership and subordinates’ OCBE through their moral identity.

Hypothesis 6: Individualism moderates the indirect relationship between responsible leadership and subordinates’ OCBE through their moral identity, such that the positive indirect relationships become stronger when individualism is low than when it is high.

## Method

### Sample and Procedure

To test our hypothesis, our primary sample was comprised of full-time subordinates from the manufacturing, real estate, and bank industries in three Chinese cities. Multiple industries were used to avoid contextual constraints associated with any particular organization. Data were collected through a Web-based survey process divided into three steps. In the first step, we contacted company managers and assured them that the survey was for academic purposes and anonymous and did not involve the company’s confidential information, and no information about the company would be leaked or shared. Simultaneously, we promised to provide to the company any feedback on valuable information obtained from an analysis of the survey responses. In the second step, we communicated with the company manager to identify a coordinator at each company. Then, the purpose of the questionnaire and the survey process were introduced to the company manager, and he or she was assured that the questionnaire would not reveal any personal information. Finally, the company manager was asked to ensure the authenticity of the survey responses and to encourage subordinates to actively participate in the survey. The third step was to send the online questionnaire to the coordinator and have the coordinator share it with the subordinates. After all of the subordinates completed the survey, the collected data were sorted and analyzed to form the final dataset.

After excluding 33 non-responses, we judged the final data, determined that 32 questionnaires with incomplete or completely inconsistent information were invalid, and finally obtained data validity matching by deleting these invalid data. A total of 273 complete and usable questionnaires were obtained out of the 338 distributed surveys, yielding an overall response rate of 80.8%. Following the 50-response threshold for each latent variable rule of thumb (Pedhazur and Schmelkin, 1991; Ababneh, 2021), the sample size of 273 is adequate for data analysis. Among the 273 participants, 41% were male, 53.8% were between 25 and 35 years old, 51.3% received a bachelor’s education, 24.5% received a postgraduate education or higher, 56% had job tenure of 3 years or shorter, and 22.3% had job tenure between 3 and 5 years. Among all of the industries, 38.8% were state-owned companies, and 40.3% were private companies. The demographic characteristics of survey samples are reported in Table 1.

**TABLE 1:** Demographic characteristics of survey sample.

	Frequency	Percent (%)
**Gender**		
Male	112	41%
Female	161	59%
**Age**		
25 years old or below	100	36.6%
25–35 years old	147	53.8%
35–45 years old	17	6.2%
45 years old or above	9	3.3%
**Education level**		
High school or below	16	5.9%
Associate’s degree	50	18.3%
Bachelor’s degree	140	51.3%
Postgraduate degree or above	67	24.5%
**Job tenure**		
3 years or below	153	56%
3–5 years	61	22.3%
5–10 years	30	11%
10 years or above	29	10.6%
**Unit character**		
State-owned companies	106	38.8%
Private companies	110	40.3%
Foreign-owned companies	13	4.8%
Other companies	44	16.1%

*N = 273.*

### Measures

Unless otherwise indicated, all variables were measured using 5-point Likert-type scales between 1 = strongly disagree and 5 = strongly agree, and all materials were presented in the Chinese language. English items were translated into Chinese following standard back-translation procedures (Brislin, 1986).

#### Responsible Leadership

We used the five-item measure by Voegtlin (2011) to assess responsible leadership. Sample items included “My direct supervisor demonstrates awareness of the relevant stakeholder claims” and “My direct supervisor considers the consequences of decisions for the affected stakeholders.” Cronbach’s alpha was 0.84 for this scale.

#### Organizational Citizenship Behavior for the Environment

We used the 10-item measure by Boiral and Paillé (2012) to assess organizational citizenship behavior for the environment. Sample items included “In my work, I weigh the consequences of my actions before doing something that could affect the environment” and “I actively participate in environmental events organized in and/or by my company.” Cronbach’s alpha was 0.92 for this scale.

#### Moral Identity

We used the 10-item measure by Aquino and Reed (2002) to assess moral identity. Sample items included “I strongly desire to have these characteristics” and “Being someone who has these characteristics is an important part of who I am.” Cronbach’s alpha was 0.90 for this scale.

#### Individualism

We used the 7-item measure by Hooft and Jong (2009) to assess individualism. Sample items included “I tend to do my own thing, and others in my family do the same” and “It is important to me that I perform better than others on a task.” Cronbach’s alpha was 0.85 for this scale.

#### Control Variables

According to previous research (e.g., Tsui and O’Reilly, 1989; Anderson and Bateman, 2000), individual demographic characteristics has potential association with outcomes such as work attitudes and behaviors. As such, gender, age, education level, job tenure and unit character served as our primary control variables. Gender was coded as: 1 = male, 2 = female. Age was coded as: 1 = 25 or less, 2 = 25–35, 3 = 35–45, 4 = 45 or above. Education level was coded as: 1 = high school or below, 2 = associate’s degree, 3 = bachelor’s degree, 4 = postgraduate degree or above. Job tenure was coded as: 1 = 3 years or less, 2 = 3–5 years, 3 = 5–10 years, 4 = 10 years or above. Unit character was coded as: 1 = state-owned companies, 2 = private companies, 3 = foreign-owned companies, 4 = other companies.

### Analysis

**FIGURE 2:**
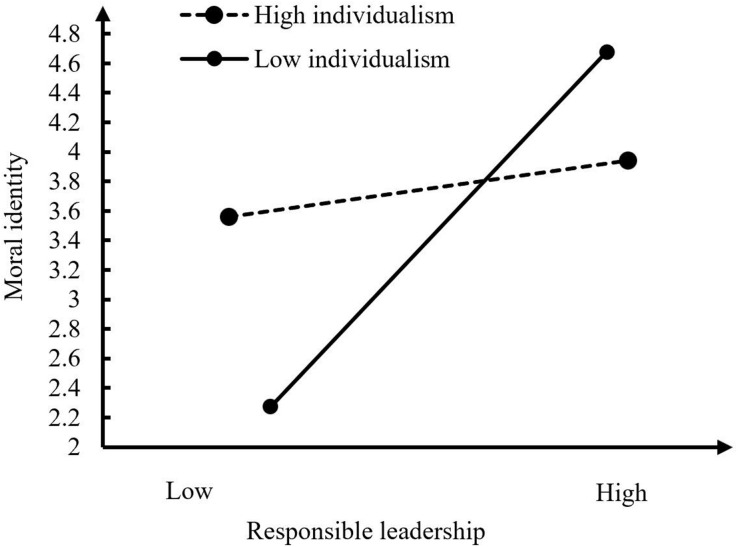
Individualism moderates the effect of responsible leadership on moral identity.

A series of confirmatory factor analyses were first conducted to confirm the discriminant validity of subordinate self-assessment variables involved in the theoretical model. In addition, descriptive statistical analysis was carried out on the variables in this study to reveal the correlation among the variables and further preliminarily verify the theoretical model. Then, we employed the causal steps described by Baron and Kenny (1986) to evaluate the mediating role of moral identity on the relationship between responsible leadership and OCBE. Although the causal steps strategy is the most commonly used method for assessing mediation, some argue that a significant total effect of an independent variable (i.e., responsible leadership) on a dependent variable (i.e., OCBE) is unnecessary (Mackinnon et al., 2000). Therefore, we used the bootstrap approach to evaluate the mediating role of moral identity, which is more powerful than the causal step procedure for small samples (Preacher and Hayes, 2008). In addition, we applied hierarchical regression analyses to evaluate the moderating role of individualism for the relationship between responsible leadership and the mediation variable (i.e., moral identity). All predictors were mean-centered to reduce the multicollinearity among the variables in the regression equation (Aiken and West, 1991). Figure 2 presents the interaction pattern for strong and weak individualism, defined as one standard deviation higher and lower than the mean value, respectively (Aiken and West, 1991). Finally, the bootstrapping-based moderated path analysis approach was applied to examine the moderated mediation hypothesis (Edwards and Lambert, 2007), which address the shortcomings of Baron and Kenny’s (1986) moderated causal steps approach and more clearly delineate the moderated and mediated nature of the relationships among the research variables (Liu et al., 2012). Analyses were conducted for the conditional indirect effects of responsible leadership on OCBE through the mediation variable (i.e., moral identity) for strong and weak individualism, respectively.

## Results

### Descriptive Statistics and Confirmatory Factor Analysis

As shown in Table 2, the CFA results demonstrate that our hypothesized four-factor model (responsible leadership, OCBE, moral identity, individualism) was a better fit into the data [χ^2^ (273) = 996.19, comparative fit index (CFI) = 0.89, Tucker-Lewis index (TLI) = 0.88, root mean square error of approximation (RMSEA) = 0.07, standardized root mean residual (SRMR) = 0.06] than alternative three-factor, two-factor, and one-factor models. Thus, we treated the four variables as independent constructs in further analyses. Table 3 depicts standardized loadings, AVE, and CR for all constructs. The composite reliability (CR) threshold of 0.60 was observed for every construct. The average variance extracted (AVE) met the recommended threshold of 0.50, with the exception of the individualism construct which showed an AVE of 0.47, only slightly lower the recommended threshold (Barbarossa et al., 2017). The means, standard deviations, and correlations among variables are reported in Table 4.

**TABLE 2:** Results of confirmatory factor analysis.

Model	Description	χ^2^	*df*	CFI	TLI	RMSEA	SRMR
Four-factor model	RL; OCBE; MI; I	996.19	458	0.89	0.88	0.07	0.06
Three-factor model 1	OCBE; I; RL and MI were combined into one factor	1335.57	461	0.81	0.80	0.08	0.07
Three-factor model 2	OCBE; MI; RL and I were combined into one factor	1771.57	461	0.72	0.70	0.10	0.11
Three-factor model 3	OCBE; RL; MI and I were combined into one factor	2337.94	461	0.60	0.57	0.12	0.22
Three-factor model 4	I; MI; RL and OCBE were combined into one factor	1336.51	461	0.81	0.80	0.08	0.07
Three-factor model 5	RL; MI; I and OCBE were combined into one factor	1777.26	461	0.72	0.70	0.10	0.11
Three-factor model 6	RL; I; MI and OCBE were combined into one factor	1619.78	461	0.75	0.73	0.10	0.08
Two-factor model 1	RL; I, MI and OCBE were combined into one factor	2401.01	463	0.59	0.56	0.12	0.12
Two-factor model 2	I; RL, MI and OCBE were combined into one factor	1913.36	463	0.69	0.67	0.11	0.10
Two-factor model 3	MI; I, RL and OCBE were combined into one factor	2114.48	463	0.65	0.62	0.11	0.12
Two-factor model 4	OCBE; I, RL and MI were combined into one factor	2114.43	463	0.65	0.62	0.11	0.12
One-factor model	RL, OCBE, MI and I were combined into one factor	2691.44	464	0.53	0.49	0.13	0.13

*N = 273.*

*CFI, comparative fit index; TLI, Tucker–Lewis index; RMSEA, root mean square error of approximation; SRMR, standardized root mean residual; RL, responsible leadership; MI, moral identity; I, individualism.*

**TABLE 3:** Factor analysis results.

Items	Standardized loadings	CR	AVE
**Responsible leadership**		0.84	0.51
My direct supervisor demonstrates awareness of the relevant stakeholder claims.	0.70		
My direct supervisor considers the consequences of decisions for the affected stakeholders.	0.80		
My direct supervisor involves the affected stakeholders in the decision-making process.	0.65		
My direct supervisor weighs different stakeholder claims before making a decision.	0.76		
My direct supervisor tries to achieve a consensus among the affected stakeholders.	0.67		
**OCBE**		0.92	0.55
In my work, I weigh the consequences of my actions before doing something that could affect the environment.	0.62		
I voluntarily carry out environmental actions and initiatives in my daily work activities.	0.70		
I make suggestions to my colleagues about ways to protect the environment more effectively, even when it is not my direct responsibility.	0.76		
I actively participate in environmental events organized in and/or by my company.	0.77		
I stay informed of my company’s environmental initiatives.	0.80		
I undertake environmental actions that contribute positively to the image of my organization.	0.73		
I volunteer for projects, endeavors or events that address environmental issues in my organization.	0.77		
I spontaneously give my time to help my colleagues take the environment into account in everything they do at work.	0.79		
I encourage my colleagues to adopt more environmentally conscious behavior.	0.74		
I encourage my colleagues to express their ideas and opinions on environmental issues.	0.72		
**Moral identity**		0.91	0.50
It would make me feel good to be a person who has these characteristics.	0.71		
Being someone who has these characteristics is an important part of who I am.	0.63		
I often wear clothes that identify me as having these characteristics.	0.84		
I would be ashamed to be a person who has these characteristics. (R)	0.70		
The types of things I do in my spare time (e.g., hobbies) clearly identify me as having these characteristics.	0.65		
The kinds of books and magazines that I read identify me as having these characteristics.	0.73		
Having these characteristics is not really important to me. (R)	0.68		
The fact that I have these characteristics is communicated to others by my membership in certain organizations.	0.70		
I am actively involved in activities that communicate to others that I have these characteristics.	0.73		
I strongly desire to have these characteristics.	0.65		
**Individualism**		0.86	0.47
I tend to do my own thing, and others in my family do the same.	0.98		
It is important to me that I perform better than others on a task.	0.66		
I am unique, different from others in many respects.	0.67		
I like my privacy.	0.63		
I would rather work alone than do a group task.	0.61		
I like to live my life independent of others.	0.61		
If I have a difficult personal problem, I rather decide by my self than consult with others.	0.59		

*N = 273.*

*OCBE, organizational citizenship behavior for the environment; CR, composite reliability; AVE, average variance extracted.*

**TABLE 4:** Means, SDs, and correlations.

Variables	*M*	*SD*	1	2	3	4	5	6	7	8	9
(1) Age	1.76	0.71	–								
(2) Gender	1.59	0.49	0.02	–							
(3) Education level	2.94	0.81	−0.18**	0.11*	–						
(4) Job tenure	1.76	1.02	0.72**	–0.05	−0.37**	–					
(5) Unit character	1.98	1.04	–0.08	0.03	–0.08	–0.07	–				
(6) Responsible leadership	3.66	0.72	0.01	0.01	–0.06	0.05	0.23**	(0.84)			
(7) OCBE	3.84	0.67	0.19**	0.10	–0.06	0.21**	0.12	0.48**	(0.92)		
(8) Moral identity	3.71	0.57	0.00	–0.06	0.07	0.11*	0.03	0.46**	0.56**	(0.90)	
(9) Individualism	3.04	0.70	–0.03	0.02	0.07	–0.08	–0.07	–0.06	–0.02	0.00	(0.85)

*N = 273.*

*SD, standard deviation.*

*Bracketed values on the diagonal are the Cronbach’s alpha value of each scale.*

*p < 0.05; **p < 0.01.

### Hypothesis Testing

Table 5 presents the hierarchical regression results of the mediation effect. In the first step, responsible leadership was positively correlated with OCBE (*B* = 0.43, *SE* = 0.05, *p* < 0.001, 95% CI[0.32,0.54] excluding zero; see Model 6), providing support for Hypothesis 1. Then, responsible leadership was positively correlated with moral identity (*B* = 0.38, *SE* = 0.04, *p* < 0.001, 95% CI[0.26,0.48] excluding zero; see Model 2), providing support for Hypothesis 2. OCBE was positively correlated with moral identity (*B* = 0.66, *SE* = 0.06, *p* < 0.001, 95% CI[0.53,0.79] excluding zero; see Model 7), Hypothesis 3 therefore received support. At the same time, supporting the second and third steps. In the fourth step, both responsible leadership and mediation variable (i.e., moral identity) were included in Model 8. The estimation results showed that moral identity (*B* = 0.52, *SE* = 0.06, *p* < 0.001, 95% CI[0.37,0.67] excluding zero; see Model 8) was still significantly related to OCBE, but the relationship of responsible leadership with OCBE was significant and positive with a reduced magnitude (*B* = 0.23, *SE* = 0.05, *p* < 0.001, 95% CI[0.10,0.37] excluding zero; see Model 8). Therefore, moral identity partially mediated the effect of responsible leadership on OCBE, providing support for Hypothesis 4. In addition, the bootstrap results indicate that the indirect effect of responsible leadership on OCBE through moral identity (*B* = 0.18, *p* < 0.01, 95% CI [0.09,0.32] excluding zero) was significant.

**TABLE 5:** Regression results.

Variable	Moral identity							OCBE						
	Model 1	Model 2		Model 3		Model 4		Model 5	Model 6		Model 7		Model 8	
	***B* (*SE*)**	***B* (*SE*)**	**95% CI**	***B* (*SE*)**	**95% CI**	***B* (*SE*)**	**95% CI**	***B* (*SE*)**	***B* (*SE*)**	**95% CI**	***B* (*SE*)**	**95% CI**	***B* (*SE*)**	**95% CI**
Age	–0.14* (0.07)	–0.13* (0.06)	[–0.24, –0.02]	–0.13* (0.06)	[–0.24, –0.02]	–0.14* (0.06)	[–0.24, –0.02]	0.06 (0.08)	0.07 (0.07)	[–0.07, 0.24]	0.15* (0.07)	[0.02, 0.30]	0.14* (0.06)	[0.01, 0.29]
Gender	–0.06 (0.07)	–0.07 (0.06)	[–0.18, 0.05]	–0.07 (0.06)	[–0.18, 0.06]	–0.09 (0.06)	[–0.20, 0.04]	0.13 (0.08)	0.12 (0.07)	[–0.02, 27]	0.17 * (0.07)	[0.05, 0.30]	0.16 (0.06)	[0.04, 0.29]
Education level	0.11* (0.05)	0.11** (0.04)	[0.03, 0.19]	0.11** (0.04)	[0.03, 0.19]	0.11** (0.04)	[0.02, 0.18]	0.02 (0.05)	0.03 (0.05)	[–0.07, 0.12]	–0.05 (0.04)	[–0.14, 0.04]	–0.03 (0.04)	[–0.12, 0.06]
Job tenure	0.16** (0.05)	0.15** (0.05)	[0.06, 0.24]	0.15** (0.05)	[0.06, 0.25]	0.15** (0.04)	[0.05, 0.25]	0.13* (0.06)	0.10 (0.05)	[–0.03, 0.21]	0.02 (0.05)	[–0.10, 0.11]	0.03 (0.05)	[–0.09, 0.12]
Unit character	0.03 (0.03)	–0.03 (0.03)	[–0.09, 0.02]	–0.03 (0.03)	[–0.08, 0.02]	–0.03 (0.03)	[–0.08, 0.03]	0.09* (0.04)	0.02 (0.04)	[–0.05, 0.07]	0.07* (0.03)	[0.01, 0.13]	0.03 (0.03)	[–0.02, 0.09]
Independent variable														
Responsible leadership		0.38*** (0.04)	[0.26, 0.48]	0.38*** (0.04)	[0.26, 0.48]	0.38*** (0.04)	[0.27, 0.48]		0.43*** (0.05)	[0.32, 0.54]			0.23*** (0.05)	[0.10, 0.37]
Mediator variable														
Moral identity											0.66*** (0.06)	[0.53, 0.79]	0.52*** (0.06)	[0.37, 0.67]
Moderator variable														
Individualism				0.03 (0.04)	[–0.06, 0.12]	0.02 (0.04)	[–0.06, 0.10]							
Responsible leadership *Individualism						–0.17** (0.06)	[–0.30,–0.02]							
*F*	2.68*	15.85***		13.62***		13.23***		4.34***	16.93***		26.62***		27.51***	
*R* ^2^	0.05	0.26		0.27		0.29		0.08	0.28		0.38		0.42	
Δ*R*^2^	—	0.21		0.01		0.02		—	0.20		0.10		0.04	

*N = 273.*

*B, unstandardized regression coefficients; *SE*, standard errors; CI, confidence interval.*

*The analysis was based on 1,000 resamples. The square brackets contain 95% confidence intervals.*

*p < 0.05; **p < 0.01; ***p < 0.001.

The interaction between responsible leadership and individualism was significant and negatively correlated with moral identity (*B* = –0.17, *SE* = 0.06, *p* < 0.01, 95% CI[–0.30,–0.02] excluding zero; see Model 4). As shown in Figure 2, the results of simple slope test indicated that the relationship between responsible leadership and moral identity was stronger when individualism was low (*simple slope* = 0.75, *p* < 0.001) than when it was high (*simple slope* = 0.10, *ns*), providing support for Hypothesis 5. Based on 1,000 resamples, the results of Table 6 further revealed that the indirect effect of responsible leadership on OCBE through moral identity was stronger when individualism was low (*B* = 0.25, *p* < 0.001, 95% CI [0.15, 0.37]) than when it was high (*B* = 0.12, *p* < 0.001, 95% CI [0.04, 0.21]). Overall, the difference between the indirect effects was also significant (*B* = –0.13, *p* < 0.01, 95% CI [–0.24,–0.03]), providing support for Hypothesis 6. The summary of hypothesis testing results is shown in Table 7.

**TABLE 6:** Results of the moderated mediation path analysis.

	First stage (P_*MX*_)		Direct effect (P_*YX*_)		Indirect effect (P_*MX*_ × P_*YM*_)	
	Estimate	Bias-corrected 95% CI	Estimate	Bias-corrected 95% CI	Estimate	Bias-corrected 95% CI
Responsible leadership→moral identity→OCBE path						
Low individualism	0.50***	[0.32, 0.61]	0.29**	[0.07, 0.49]	0.25***	[0.15, 0.37]
High individualism	0.25***	[0.09, 0.37]	0.23**	[0.07, 0.39]	0.12***	[0.04, 0.21]
Difference between low and high	–0.25*	[–0.41, –0.03]	–0.06	[–0.26, 0.25]	–0.13**	[–0.24, –0.03]

*N = 273.*

*CI, confidence interval; OCBE, organizational citizenship behavior for the environment.*

*The analysis was based on 1,000 resamples. The square brackets contain bias-corrected 95% confidence intervals. P_*MX*_, Path from the independent variable (i.e., responsible leadership) to mediator (i.e., moral identity); P_*YM*_, Path from the mediator (i.e., moral identity) to dependent variable (i.e., OCBE); P_*YX*_, Path from the independent variable (i.e., responsible leadership) to the dependent variable (i.e., OCBE).*

*p < 0.05; **p < 0.01; ***p < 0.001.

**TABLE 7:** Summary of hypothesis testing results.

	Hypotheses	Findings
Hypothesis 1	Responsible leadership is positively related to subordinates’ OCBE.	Supported
Hypothesis 2	Responsible leadership is positively related to subordinates’ moral identity.	Supported
Hypothesis 3	Subordinates’ moral identity is positively related to subordinate’s OCBE.	Supported
Hypothesis 4	Subordinates’ moral identity mediates the relationship between responsible leadership and subordinates’ OCBE.	Supported
Hypothesis 5	Individualism moderates the relationship between responsible leadership and subordinates’ moral identity, such that the relationship is weaker when individualism is high rather than low.	Supported
Hypothesis 6	Individualism moderates the indirect relationship between responsible leadership and subordinates’ OCBE through their moral identity, such that the positive indirect relationships become stronger when individualism is low than when it is high.	Supported

*N = 273.*

*OCBE, organizational citizenship behavior for the environment.*

## Discussion

Drawing on social identity theory (Tajfel and Turner, 1979), we developed and tested a model explaining how and when engaging in responsible leadership affects subordinates’ OCBE. Responsible leadership was found to be positively related to subordinates’ moral identity, which in turn was positively related to subordinates’ OCBE. Subordinates’ moral identity partially mediated the relationship between responsible leadership and subordinates’ OCBE. In addition, both the relationship between responsible leadership and subordinates’ moral identity and the indirect relationship between responsible leadership and subordinates’ OCBE were stronger with lower individualism.

### Theoretical Implications

Our findings offer several important theoretical implications. First, from the social identity perspective, this research provides a novel perspective on how responsible leadership enhances subordinates’ OCBE. Although the OCBE literature has highlighted the critical role of leadership on the emergence of OCBE (e.g., Choi, 2007; Ramus and Killmer, 2007; Daily et al., 2008; Paillé et al., 2013a; Han et al., 2019), considerably less is known about the underlying psychological processes and mechanism though which leaders motivate subordinates’ OCBE. Social identity theory suggests that subordinates’ moral identity is a central component that shapes their ethical behaviors (Tajfel and Turner, 1979; Ashforth and Mael, 1989; May et al., 2015). Nevertheless, previous research has yet to determine whether leaders influence subordinates’ OCBE by shaping their moral identity. To fill this gap, this study was based on social identity theory and focused on the mediating role of subordinates’ moral identity to determine the effect of responsible leadership on their OCBE, whereas the extant literature typically relied on social exchange theory (e.g., Paillé et al., 2013a,b), social learning theory (e.g., Han et al., 2019), planned behavior theory (e.g., Greaves et al., 2013), deontic justice theory (e.g., Erdogan et al., 2015), self-determination theory (e.g., Graves et al., 2013), and developmental theory (e.g., Boiral et al., 2016) to account for the occurrences of OCBE. Subordinates’ moral identity was found to be an important explanatory mechanism for transmitting the effects of responsible leadership on their OCBE. Consistent with social identity theory, responsible leaders who attach importance to the interests of environmental stakeholders and corporate moral values are likely to evoke moral identity among their subordinates. In turn, subordinates with a strong moral identity regard moral values as the center of their self-definition and tend to engage in ethical behaviors that fit with organizational moral values, such as OCBE.

Second, this study extends the current understanding of the boundary conditions under which responsible leadership is effective or ineffective, by demonstrating individualism as a key moderator. Based on the cultural value perspective, this study found that the extent to which responsible leadership promoted subordinates’ moral identity depended on the cultural value (i.e., individualism) factor. Specifically, subordinates with strong individualism pay attention to the realization of personal goals, follow self-values, and are independent of other members of the organization, which may conflict with corporate goals and organizational values advocated by responsible leadership. In contrast, subordinates with weak individualism tend to adapt to the goals and values of their leadership or organization, making responsible leadership significantly effective in helping subordinates shape and improve their moral identity. This finding suggests that solely displaying responsible leadership behavior is not enough to induce subordinates to enhance their moral identity, but it is also contingent on the discrepancies of individual cultural values, such as the degree of individualism. In other words, not all subordinates are motivated by their responsible leaders. Therefore, this research provides new insights into comprehending how to improve the effectiveness of responsible leadership on subordinates’ moral identity and answers the important question of a call for investigating the influence of individualism cultural values in an organization (Hooft and Jong, 2009; Santos et al., 2017).

Third, this study’s examination of the integrated model indicated that responsible leadership offers stronger benefits toward enhancing subordinates’ OCBE through their moral identity if such subordinates have weak individualism, thereby providing a clear picture for understanding how responsible leadership influences OCBE and how such influence is shaped by subordinates’ individualism. Specifically, responsible leadership views upholding the interests of environmental stakeholders as the goal of corporations, because doing so creates a positive moral and cultural atmosphere and involves subordinates in the organizational decision-making process, which are consistent with the cultural values of weak individualism. Therefore, such consistency enhances subordinates’ OCBE by strengthening their moral identity in the context of the cultural values of weak individualism. This finding suggests that in the context of cultural values of weak individualism, the effect of responsible leadership on subordinates’ OCBE through the moral identity is stronger than in the context of the cultural values of strong individualism. In other words, the mediating role of subordinates’ moral identity shows a significant discrepancy under the background of different levels of individual cultural values. The results of this study clearly indicated that subordinates with a certain type of cultural values have stronger moral to responsible leaders. Concurrently, this finding positively addresses the call of researchers to pay more attention to the impact of subordinates’ values on the relationship between responsible leadership and their OCBE (Han et al., 2019).

### Practical Implications

This research offers several implications for practice. First, the findings in this study suggest that responsible leadership can be useful in facilitating subordinates’ moral identity and OCBE. Indeed, to enable organizations to achieve sustainable development in an ever-changing marketplace, previous research has begun to call for teaching managers to be responsible leaders (Doh and Quigley, 2014; Antunes and Franco, 2016). Therefore, organizations should train their current leaders to develop responsible leaders. For example, organizations can help leaders clarify their roles and behaviors by formulating explicit rules of duty (Maak and Pless, 2006) that are combined with the goals of organizational development, conducting courses on supervisors’ sense of responsibility (Voegtlin et al., 2012), and establishing a long-term learning mechanism to cultivate and develop the comprehensive leadership ability of responsible leaders (Maak et al., 2016). In addition, when recruiting members and selecting leaders, the human resource management department can determine relevant assessment standards, comprehensively evaluate their cognitive abilities, values, and moral quality (Pless, 2007; Maak et al., 2016), and further assess their consistency using organizational values (Liu and Lin, 2017). Doing so can assist organizations in identifying the personnel with the potential for responsible leadership.

Second, this research presents subordinates’ moral identity as a bridge that links responsible leadership to their OCBE. Hence, selecting subordinates who display moral identity could present an avenue for enhancing OCBE in a responsible organization. The extant literature has begun to focus on the importance of behaving ethically and improving the moral identity of subordinates in an organization (Trevino et al., 2014). On the one hand, leaders should care about and restrain unethical behavior in the organization, support subordinates’ ethical behavior, attach importance to subordinates’ opinions, and involve them in participating in organizational decision making to increase their moral identity (Doh and Quigley, 2014). On the other hand, organizations need to establish moral norms and require its members to be responsible for unethical behavior (Kaptein, 2010), provide moral training to improve subordinates’ moral cognition (Mulder and Aquino, 2013), and form a moral organizational culture to cultivate their moral values to attract ethical applicants to join the organization during the recruiting process and to retain subordinates who value ethics (Rupp et al., 2013; May et al., 2015). In addition, according to social identity theory (Tajfel and Turner, 1979; Tajfel, 1982), when employees have a positive social identity with the group and organization they belong to, they will improve their attitudes and behaviors to align with the organization’s behavioral norms, values and goals. Therefore, managers need to emphasize and enhance employees’ inter group identity in the organization, such as creating a label like “our team” in the organizational culture, trying to incorporate the concept of inter group identity into important management practices, and incorporating inter group identity into performance evaluations and incentive programs to further increase employees’ motivation for OCBE (Aquino and Reed, 2002; May et al., 2015).

Finally, the positive effect of responsible leadership in enhancing subordinates’ OCBE through their moral identity was found to be weaker for subordinates with strong individualism. This finding suggests that an organization’s responsible leaders should be aware that their behavior may lead to different reactions depending on their subordinates’ individual cultural values. From the perspective of contingency, leaders need to flexibly take corresponding measures with subordinates with different degrees of individualism. Therefore, to better match the responsible leadership, corresponding measures need to be taken to judge the degree of subordinates’ individualism. Leaders may be trained to infer the degree of individualism of their subordinates by observing their behaviors. During the recruitment process, a systematic assessment can be carried out through personal tests to better understand the degree of individualism of the candidates. Leaders can then use such information to adjust their subordinates’ coping strategies with different degrees of individualism to ensure that responsible leadership can generate stronger moral identity among them. In addition, for subordinates with strong individualism, organizations need to set interdependent tasks to improve their cooperative behavior (Earley, 1989; Wagner, 1995) and establish an organizational culture of cooperation and sharing to reduce the degree of individualism (Morris et al., 1993).

### Limitations and Future Research Directions

This research has several limitations that should be noted. First, our research may lead to common method biases because the data were collected from the same source. Although confirmatory factor analysis shows that the variables have good discriminate validity, future research is encouraged to collect data through the mutual evaluation of supervisors and subordinates at different times. Concurrently, because this research is cross-sectional by design, causality cannot accurately be inferred. For example, it is possible that when subordinates have higher awareness of OCBE, their moral identity may be strengthened. Hence, although the suggested patterns of causality seem plausible using the current theorizing and data, further experimental or longitudinal research is required to replicate and extend this study’s research model in the future. In addition, the current research was based on subordinate self-reported data in a cross-sectional survey, which is prone to social desirability bias. While it appears on the surface that self-report measures might be biased by social desirability, previous empirical research reveals that social desirability has a low or nil effect on the way people report their pro-environmental behaviors in anonymous questionnaires (Milfont, 2009; Kormos and Gifford, 2014). Simultaneously, the use of self-report measures in the current research is particularly justified because subordinates may be more aware of their own behavior compared to supervisors and coworkers who may not have the opportunity to accurately observe subordinates’ behavior (Norton et al., 2017). Nevertheless, future research could include other more objective measures or attempt to collect other people’s ratings of OCBE to avoid social desirability bias.

Second, although a social identity theory relevant mediator (i.e., moral identity) was examined and its effect was simultaneously tested, other theoretical mechanisms can still help explain the relationship between responsible leadership and subordinates’ OCBE, such as social exchange theory (e.g., Paillé et al., 2013a,b), social learning theory (e.g., Han et al., 2019), and planned behavior theory (e.g., Greaves et al., 2013). Future research should provide a more detailed theoretical model to test the effect of other mechanisms and to control these mechanisms simultaneously to discover the strength and unique contributions of the current research mechanisms. In addition, regulatory focus theory holds that the self-regulatory focus of subordinates is a core component that affects their motivations and behaviors (Yang et al., 2018). However, research has yet to determine whether responsible leadership influences subordinates’ OCBE by enhancing their promotion focus. Thus, further clarifying the internal mechanism of responsible leadership and subordinates’ OCBE from a novel theoretical perspective is advocated.

Third, this study adopted the perspective of cultural values and used individualism as a crucial boundary condition to understand the circumstances under which responsible leadership will be strengthened or weakened. This understanding can assist in a better understanding of changes in cultural values in the context of world economic globalization. Hence, future research is encouraged to identify in a clearer manner the impact of other cultural values on hypothetical relationships, such as traditionality (Farh et al., 1997) and power distance (Der Vegt et al., 2005). Moreover, to better explain the unique contribution of individualism as a boundary condition, future research should control the existing moderating variables related to cultural values, such as supervisor-subordinate guanxi (Han et al., 2019).

Fourth, although this research has supported the significance of responsible leadership from the theory related to responsible leadership, some conceptual overlaps exist among the elements of responsible leadership and other leadership structures, such as ethical leadership (Brown et al., 2005), transformational leadership (Rafferty and Griffin, 2004), and servant leadership (Chen et al., 2015). However, during the hypotheses testing process, these structures were not controlled for in this study. This area is key for future research because it can test whether responsible leadership can explain additional unique variances and the reasons for such differences.

Finally, despite our sample size being comparable to other studies published recently in top tier journals (e.g., Zientara and Zamojska, 2018, *N* = 239; Pham et al., 2019, *N* = 203; Kesenheimer and Greitemeyer, 2021, *N* = 261; Zhao et al., 2021, *N* = 302), a potential limitation is the relatively small sample size of the current research, which may limit the generalizability of the results. Meanwhile, our primary sample was comprised of full-time subordinates from the manufacturing, real estate, and bank industries. Although multiple industries were used to avoid contextual constraints associated with any particular organization, we may have overlooked the fact that the importance of subordinates’ OCBE in hospitality and tourism is more acute because of the sector’s reliance on the attractiveness of the natural environment (Kim et al., 2016; Rezapouraghdam et al., 2018). In addition, because the data used in this study were collected from Chinese samples, the degree of individualism of individuals in the sample is lower relative to that of Westerners. Indeed, cultural characteristics may have affected the research results in this study. Cultures characterized by strong individualism, such as the United States (Earley, 1993), pay more attention to individual independence and freedom. Therefore, the relational model developed in this study may be less prominent in such a cultural context. Although empirical research has been carried out in the Chinese context and has shown that individualism significantly affects the role of responsible leadership, extending these findings to other cultural contexts in the future needs to be done cautiously. Thus, the generalizability of the current findings could be further enhanced by testing the current model in other institutional contexts. Therefore, we encourage future scholars to collect larger samples of data in other institutional contexts and for more representative sectors like hospitality and tourism to further validate the findings of this research.

## Conclusion

To ensure that organizations obtain competitive advantages and sustainable developments in an ever-changing marketplace, understanding how and when responsible leadership enhances subordinates’ OCBE has become important. The current research extends the cognition about the relationship between responsible leadership and subordinates’ OCBE by exploring the mediating role of their moral identity and the moderating role of individualism. The findings in this study not only confirm the effectiveness of responsible leadership in the organization but also highlight its boundary conditions for facilitating subordinates’ OCBE. We hope that the theoretical insights gained through this effort will spur further research aimed at the antecedents of subordinates’ OCBE.

## Data Availability Statement

The raw data supporting the conclusions of this article will be made available by the authors, without undue reservation.

## Author Contributions

XX and FY: conceptualization. ZZ: methodology, formal analysis, writing—original draft preparation, and writing—review and editing. HQ: investigation and data curation. FY: visualization and supervision. XX: project administration. All authors: read and agreed to the published version of the manuscript.

## Conflict of Interest

The authors declare that the research was conducted in the absence of any commercial or financial relationships that could be construed as a potential conflict of interest.

## Publisher’s Note

All claims expressed in this article are solely those of the authors and do not necessarily represent those of their affiliated organizations, or those of the publisher, the editors and the reviewers. Any product that may be evaluated in this article, or claim that may be made by its manufacturer, is not guaranteed or endorsed by the publisher.
